# Microstructural Change Due to Aging and Its Effect on Fatigue Properties in Sn-Sb-Ag-Ni-Ge Alloy

**DOI:** 10.3390/ma19132710

**Published:** 2026-06-24

**Authors:** Kohei Mitsui, Hirohiko Watanabe, Kosuke Kimura, Ikuo Shohji

**Affiliations:** 1 Fuji Electric Co., Ltd., Hino 1910062, Japan; 2 Toray Research Center, Inc., Ohtsu 5208567, Japan; kosuke.kimura.h3@trc.toray; 3 Graduate School of Science and Technology, Gunma University, Kiryu 3768515, Japan; shohji@gunma-u.ac.jp

**Keywords:** Sn-Sb-Ag-Ni-Ge, Ag_3_Sn, Ostwald ripening, microstructure, low cycle fatigue, power semiconductor

## Abstract

In this study, the microstructural changes and coarsening behavior of Ag_3_Sn in Sn-6.4Sb-3.9Ag-0.25Ni-0.003Ge (mass%) during high-temperature aging were investigated. Additionally, low-cycle fatigue tests were conducted to compare the fatigue behavior of Sn-6.4Sb-3.9Ag-0.25Ni-0.003Ge with that of Sn-3.0Ag-0.5Cu. At room temperature, SbSn phases are dispersed in the β-Sn matrix. As the temperature rises, Sb atoms dissolve in the β-Sn phase; thus, the SbSn phases disappear, and some of the atoms aggregate. The activation energy was 45 kJ/mol for the coarsening of Ag_3_Sn in Sn-6.4Sb-3.9Ag-0.25Ni-0.003Ge due to aging. Ag_3_Sn coarsening was estimated to be controlled by the lattice diffusion of Ag atoms in the β-Sn phase. Furthermore, it was confirmed that the solid solution of Sb atoms in the β-Sn phase reduces the solubility limit of Ag atoms in the β-Sn phase, which delays the coarsening of Ag_3_Sn. Regarding fatigue properties, while both alloys exhibited comparable low-cycle fatigue behavior at room temperature, the fatigue ductility exponent’s increase was confirmed to be suppressed for the Sn-6.4Sb-3.9Ag-0.25Ni-0.003Ge alloy at 175 °C. This trend suggests that the delayed coarsening of Ag_3_Sn maintains the cyclic strain-hardening exponent, thereby influencing high-temperature fatigue behavior.

## 1. Introduction

Various additive elements have been studied to improve the properties of lead-free Sn-rich solders. Among these, Sn-Ag-Cu and Sn-Ag system solders containing Ag are widely used due to their excellent mechanical properties. Adding Ag to a solder generates Ag_3_Sn, which finely disperses within the metal structure and is stable compared with other elements, resulting in superior tensile properties and fatigue and creep resistance [1,2,3].

In recent years, demand for power electronics products has been increasing from an energy management perspective. The requirement for heat resistance in solder materials, including high-temperature fatigue properties, has also increased, making intermetallic compound reinforcement with Ag_3_Sn more important. For example, power semiconductors currently require heat resistance of 150 °C or 175 °C [4], and in the future, wide-bandgap semiconductors such as silicon carbide (SiC) and gallium nitride (GaN) will be expected to require a heat resistance of over 200 °C [5].

However, it is generally known that intermetallic compounds (IMCs) coarsen when exposed to high temperatures for long periods, resulting in a loss of dispersion density. The coarsening behavior of the microstructure in solder alloys exposed to high-temperature environments has been investigated in representative solder compositions such as Sn-Pb and Sn-Ag-Cu solders. The coarsening of Sn-Pb solder’s eutectic structure has been investigated by Jung et al. [6,7], and that of Sn-3.0Ag-0.5Cu has been studied by I. Dutta, S.L. Allen et al., and others [8,9,10,11,12,13]. However, few reports have quantitatively evaluated or elucidated the principles of compound coarsening in solder alloys other than Sn-Pb and Sn-Ag-Cu systems.

Reports indicate that the presence of solute elements in the solder effectively suppresses compound coarsening. Specifically, several reports have confirmed that adding the solid solution element Bi can suppress Ag_3_Sn coarsening in Sn-Ag-Cu solder [14,15]. However, adding Bi to Sn-rich solder raises concerns about Bi aggregates at the joint interface due to thermal aging, which can cause brittle fractures [16].

Sb is another element that exhibits a solid solution strengthening mechanism when added to Sn. At room temperature, Sb reacts with Sn to form SbSn precipitates, resulting in precipitation hardening. However, as the temperature increases, Sb dissolves into Sn. At temperatures above 100 °C, solution hardening occurs in addition to precipitation hardening [17,18]. We have focused on Sn-Sb-Ag alloys as a solder material to be applied to power semiconductors, which are expected to operate at high temperatures. Based on the ternary eutectic composition of Sn-Sb-Ag, we added trace amounts of Ni and Ge and developed Sn-6.4Sb-3.9Ag-0.25Ni-0.003Ge (mass%) to enhance heat resistance and improve productivity. In Sn-6.4Sb-3.9Ag-0.25Ni-0.003Ge, the dispersion of Ag_3_Sn, combined with the solid solution of Sb at high temperatures, is expected to suppress the coarsening of the Ag_3_Sn. While prior studies on Sn-Sb-Ag alloys have mostly evaluated high-temperature fatigue properties and performed basic microstructural observations [5,19], this study visualizes the microstructural evolution of the Sn-6.4Sb-3.9Ag-0.25Ni-0.003Ge alloy as a function of temperature and quantifies the coarsening behavior of Ag_3_Sn during aging. Furthermore, we clarify their subsequent impact on high-temperature fatigue properties.

The purpose of this study is to clarify the behavior of Sb in Sn-6.4Sb-3.9Ag-0.25Ni-0.003Ge, to visualize its microstructural evolution as a function of temperature, to investigate the effect of solute Sb on the ripening of Ag_3_Sn, and to evaluate its subsequent effects on fatigue properties.

## 2. Materials and Methods

**Figure 1:**
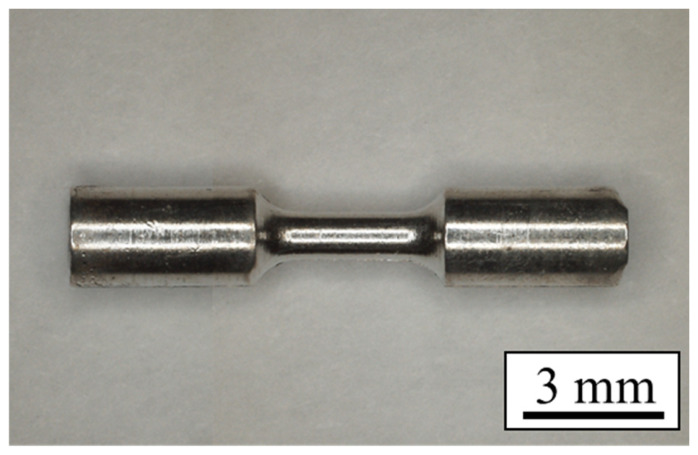
Appearance of Sn-6.4Sb-3.9Ag-0.25Ni-0.003Ge specimen.

**Figure 2:**
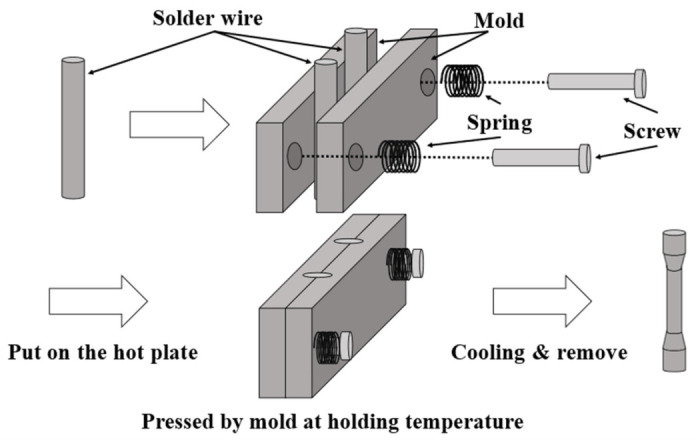
Fabrication flow of specimen.

This study focused on Sn-6.4Sb-3.9Ag-0.25Ni-0.003Ge and, as a comparative material, the widely used industrial Sn-3.0Ag-0.5Cu (mass%). The melting points of both alloys were measured using differential scanning calorimetry (DSC) according to JIS Z 3198-1 [20]. The DSC melting point measurement results are shown in Table 1. The specimens were cast using solder wire produced by NIHON HANDA Co., Ltd. (Tokyo, Japan). The casting temperature was set to the liquidus temperature of each alloy plus 30 °C. The gage length and diameter of the specimen were 2.4 mm and 1 mm, respectively. Figure 1 shows the appearance of the specimen. The fabrication flow of the specimen is shown in Figure 2. As-cast dog-bone-type specimens were fabricated with solder wire using a divided mold. The cooling rate of solidification was controlled by removing the metal mold with the specimen from the hot plate and promptly cooling it on the stainless plate. The cooling rate of Sn-6.4Sb-3.9Ag-0.25Ni-0.003Ge and Sn-3.0Ag-0.5Cu during solidification was 5 °C/s.

Aging treatments were performed on the specimens at 150 °C, 175 °C, and 200 °C for 250 h, 500 h, and 1000 h. After aging, the specimens were embedded in epoxy resin, and the cross-section was polished to investigate the microstructure. The microstructure was observed with a scanning electron microscope (SEM) (SU5000, Hitachi High-Tech Corp., Tokyo, Japan). Then, Ag_3_Sn was distinguished from other compounds using backscattered electron (BSE) images. The sizes of Ag_3_Sn particles were measured using the image processing software WinROOF2018 (MITANI Corp., Fukui, Japan), and their area equivalent diameter was defined as the particle size. For the initial microstructure, the microstructural observation was conducted with an electron probe X-ray microanalyzer (EPMA) (EPMA-1610, Shimadzu Corp., Kyoto, Japan). In addition, microstructural changes in Sn-6.4Sb-3.9Ag-0.25Ni-0.003Ge with increased temperature were observed in situ using transmission electron microscopy (TEM) (JEM-F200, JEOL Ltd., Tokyo, Japan). Low-cycle fatigue tests were conducted at a strain rate of 4.2 × 10^−4^ s^−1^ at temperatures of 25 °C and 175 °C using a microload test system (LMH207, SAGINOMIYA SEISAKUSHO, Inc., Tokyo, Japan). A symmetrical triangular waveform was used for displacement control. The total strain range, Δ*ε*_t_, varied from 0.5% to 2.0%. Fatigue life was defined as the number of cycles at which the maximum load decreased by 30% compared with its value at the fifth cycle [21]. For fatigue testing, as-cast specimens were used without any aging treatment.

## 3. Results and Discussion

### 3.1. Initial Microstructure

**Figure 3:**
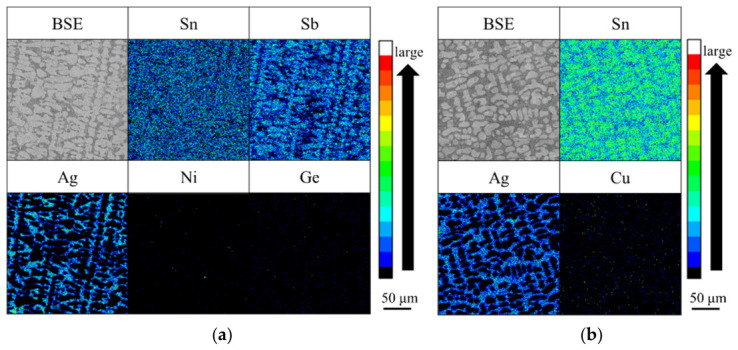
Results of mapping analysis of as-cast microstructures with EPMA: (**a**) Sn-6.4Sb-3.9Ag-0.25Ni-0.003Ge and (**b**) Sn-3.0Ag-0.5Cu.

**Figure 4:**
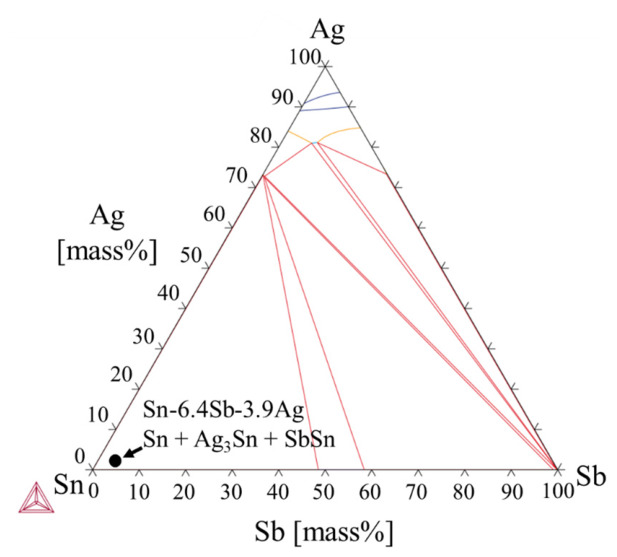
Ternary phase diagram of Sn-Sb-Ag calculated by Thermo-Calc 2024a at room temperature.

Figure 3 shows the EPMA mapping analysis results for Sn-6.4Sb-3.9Ag-0.25Ni-0.003Ge and Sn-3.0Ag-0.5Cu. For Sn-6.4Sb-3.9Ag-0.25Ni-0.003Ge, a thermodynamic diagram of the Sn-Sb-Ag ternary system was created using Thermo-Calc 2024a (Thermo-Calc Software AB, Solna, Sweden), as shown in Figure 4. In both alloys, the microstructure shows that Ag_3_Sn formed dendrites around the β-Sn matrix phase. In Sn-6.4Sb-3.9Ag-0.25Ni-0.003Ge, the locations where Sb is detected coincide with the locations where Sn is detected. Therefore, SbSn forms are inferred to be within the β-Sn dendrites in the as-cast microstructure. As shown in a TEM image, since most of the SbSn compounds are on the order of several tens of nanometers in size, they could not be confirmed in the BSE image shown in Figure 3a.

Furthermore, due to their trace amounts, both Ni and Ge were scarcely detected by EPMA mapping. In terms of their distribution, Ni dissolves into interfacial IMCs when Cu is involved in interfacial reactions [22], but it remains as a minor Sb-Ni compound within the bulk matrix [19]. Meanwhile, Ge preferentially migrates to the solder surface to exist as GeO_2_ [23]. Consequently, neither element seems to significantly influence the coarsening of Ag_3_Sn.

**Figure 5:**
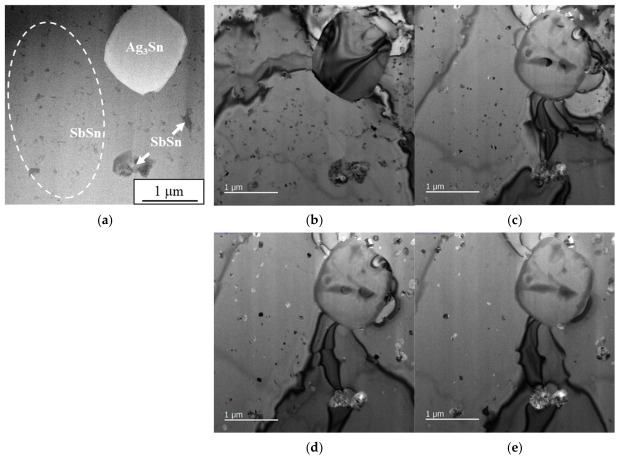
In situ TEM observations of as-cast Sn-6.4Sb-3.9Ag-0.25Ni-0.003Ge with temperature change: (**a**) HAADF image before heating, (**b**) at R.T., (**c**) at 150 °C, (**d**) at 175 °C, and (**e**) at 200 °C.

It is generally known that the solubility of Sb in the solder’s matrix phase β-Sn increases with rising temperature [18]. Figure 5 shows in situ TEM images of the initial microstructures of Sn-6.4Sb-3.9Ag-0.25Ni-0.003Ge observed before heating and during the heating process at room temperature, 150 °C, 175 °C, and 200 °C. The temperature was raised at a rate of 5 °C/min while the microstructure was observed. To prevent electron-beam damage to the in situ testing specimen, the types of intermetallic compounds were pre-identified using EDS analysis in a separate field of view; during the actual observation, each phase was distinguished based on the contrast variations in the High-Angle Annular Dark Field (HAADF) Scanning TEM images. In the HAADF image, the light-gray compound visible in the upper right corner is Ag_3_Sn, while the dark-gray particles on the order of several tens of nanometers scattered throughout the β-Sn matrix are SbSn. Due to thermally induced stress relaxation during heating, bend contours associated with local crystal bending can be observed as a faint, time-dependent stripe-like contrast within the grain. At room temperature, SbSn is finely dispersed. However, as the temperature rises, the SbSn disappears and aggregates. This is because Sb dissolves into Sn as the temperature rises, suggesting that the solubility limit of Sb in Sn-6.4Sb-3.9Ag-0.25Ni-0.003Ge increases as the temperature rises. Note that the fine SbSn disappears first, while the micrometer-order SbSn disappears more slowly than the nano-order SbSn. By contrast, within the limited duration of the in situ observation, the Ag_3_Sn did not seem to disappear or aggregate and continued to exist stably at any temperature.

### 3.2. Change in Particle Size of Ag_3_Sn Compounds Caused by Aging

**Figure 6:**
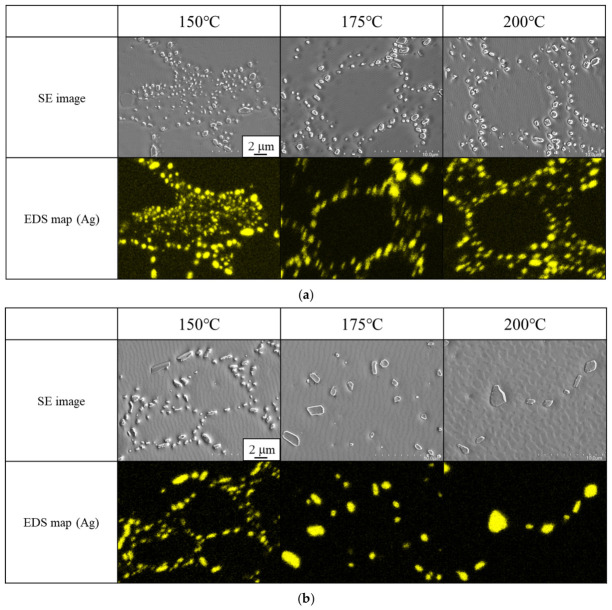
SE images of microstructures of both solders after aging for 1000 h at each temperature: (**a**) Sn-6.4Sb-3.9Ag-0.25Ni-0.003Ge; (**b**) Sn-3.0Ag-0.5Cu.

Figure 6 shows secondary electron (SE) images and a Ag EDS map of representative microstructures after aging for 1000 h at 150 °C, 175 °C, and 200 °C. As shown in Figure 3, the initial microstructures exhibit no significant differences between the microstructures of either solder alloy. However, in Sn-3.0Ag-0.5Cu, the dendrite shape of β-Sn shown in the initial microstructure (refer to Figure 3) becomes indistinct, and the dispersion of compounds becomes sparse as the aging temperature increases. By contrast, in Sn-6.4Sb-3.9Ag-0.25Ni-0.003Ge, the dendrite structure and dispersion of compounds are maintained. The compounds shown in Figure 6 can be inferred to be Ag_3_Sn based on mapping analysis. Thus, the particle size of Ag_3_Sn in the Sn-6.4Sb-3.9Ag-0.25Ni-0.003Ge alloy is smaller than that in Sn-3.0Ag-0.5Cu.

**Figure 7:**
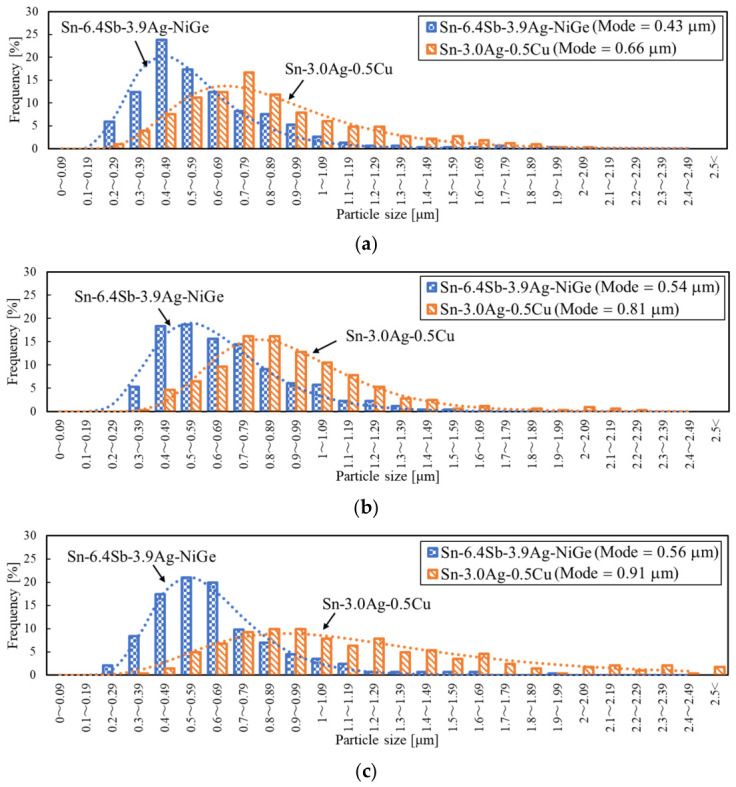
Distribution of particle size of Ag_3_Sn after isothermal aging for 1000 h: (**a**) 150 °C, (**b**) 175 °C, and (**c**) 200 °C.

Figure 7 shows histograms of the Ag_3_Sn particle sizes after aging at 150 °C, 175 °C, and 200 °C for 1000 h. Each distribution is fitted with a log-normal distribution, and the maximum value of the curve obtained by fitting is taken as the mode. Focusing on the mode, Sn-6.4Sb-3.9Ag-0.25Ni-0.003Ge tended to have a smaller Ag_3_Sn particle size than Sn-3.0Ag-0.5Cu at all heat treatment temperatures. The particle size of Ag_3_Sn of Sn-3.0Ag-0.5Cu after aging for 1000 h was approximately 1.5 to 1.7 times that of Sn-6.4Sb-3.9Ag-0.25Ni-0.003Ge.

### 3.3. Derivation of Activation Energy for Ostwald Ripening of Ag_3_Sn

The coarsening of IMCs is a process known as Ostwald ripening, during which the compound dissolves and disappears, and the material diffuses through the medium and transforms into coarse particles. Ostwald ripening follows Equation (1):

(1) $$D^{n}-{D_{0}}^{n}=K\;t\exp(-\frac{Q}{RT})$$

where *D* and *D*_0_ are the diameter of Ag_3_Sn particles at times *t* and 0, respectively. *n* is the ripening exponent, *K* is a constant, *Q* is the activation energy, *R* is the gas constant, and *T* is the absolute temperature. The ripening exponent, *n*, is associated with the diffusion mode during compound coarsening. When compound coarsening is controlled by interface reaction or lattice diffusion, *n* is 2 or 3, respectively. Since the coarsening of the Ag_3_Sn compound is controlled by the lattice diffusion of Ag under the present experimental temperature conditions based on the literature [24], *n* = 3 was adopted. By rearranging Equation (1), it can be expressed using an Arrhenius plot, as in Equation (2). In this case, the slope of the regression line in the Arrhenius plot of Equation (2) represents the activation energy when the second phase coarsens.

(2) $$\ln\;(\frac{D^{3}-{D_{0}}^{3}}{t})=-\frac{Q}{R}\times \;\frac{1}{T}+\ln\;(K)$$

**Figure 8:**
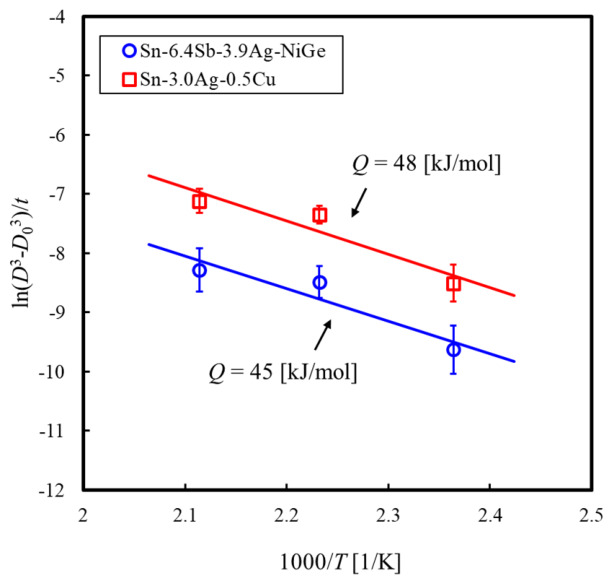
Arrhenius plot for Ostwald ripening of Ag_3_Sn in Sn-6.4Sb-3.9Ag-0.25Ni-0.003Ge and Sn-3.0Ag-0.5Cu.

**Figure 9:**
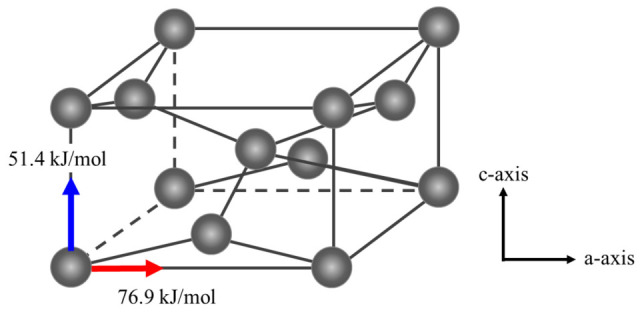
Relationship between diffusion direction and activation energy for Ag in β-Sn (adapted from [24]).

Figure 8 shows the Arrhenius plots created based on Equation (2). Given the slopes of the regression lines in Figure 8, the activation energies for Sn-6.4Sb-3.9Ag-0.25Ni-0.003Ge and for Sn-3.0Ag-0.5Cu were 45 kJ/mol and 48 kJ/mol, respectively. A previous study reported that the activation energy for Ag_3_Sn coarsening in the Sn-Ag-Cu system alloy is 50 kJ/mol; the value is close to the activation energy of 51.4 kJ/mol for diffusion parallel to the c-axis of the β-Sn lattice, as shown in Figure 9. Thus, the activation energy for the Ostwald ripening of Ag_3_Sn in Sn-6.4Sb-3.9Ag-0.25Ni-0.003Ge was close to this value. Therefore, the temperature dependence of the Ostwald ripening rate for Ag_3_Sn—namely, the activation energy, which corresponds to the slope on the Arrhenius plot—seemed to be comparable regardless of the presence of the solid solution element Sb.

**Figure 10:**
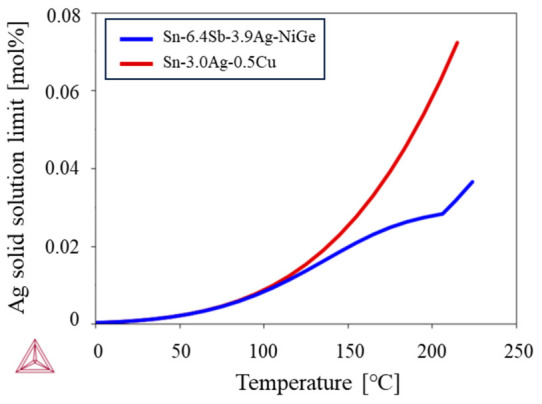
Change in solid solubility limit of Ag in each solder with temperature change.

However, the difference in coarsening behavior shown in Figure 6 may be affected by the intercept on the Arrhenius plot. This is determined by the proportionality constant, *K*, in Equation (2), as shown in Equation (3).

(3) $$K=\frac{B\gamma V_{m}C_{0}D_{sol}}{RT}$$

where B is the constant, *γ* is the specific energy of the particle–matrix interface, *V*_m_ is the molar volume of the second phase, *C*_0_ is the equilibrium–solute concentration in the matrix, and *D*_sol_ is the effective solute diffusivity in the matrix [8]. Since the solubility, *C*_0_, of Ag in β-Sn is low, Ag_3_Sn is less prone to coarsening, resulting in superior mechanical properties for Ag-added solder alloys. In Sn-6.4Sb-3.9Ag-0.25Ni-0.003Ge, Sb is solid-solubilized, further reducing the Ag solubility limit and likely affecting *K*. Figure 10 shows the Thermo-Calc results for the Ag solid solubility in Sn-6.4Sb-3.9Ag-0.25Ni-0.003Ge and Sn-3.0Ag-0.5Cu as a function of temperature. Due to the increased solid solubility of Sb in Sn at high temperatures (refer to Figure 5), the solid solubility limit of Ag in Sn-6.4Sb-3.9Ag-0.25Ni-0.003Ge is smaller than that in Sn-3.0Ag-0.5Cu. At 200 °C, the solid solubility limit of Ag in Sn-6.4Sb-3.9Ag-0.25Ni-0.003Ge is less than half that of Sn-3.0Ag-0.5Cu. This difference in the solubility of Ag in the matrix affects *K* in Equations (1) and (2), which delays the coarsening of Ag_3_Sn in Sn-6.4Sb-3.9Ag-0.25Ni-0.003Ge.

### 3.4. Effect of Differences in IMC Coarsening Behavior on Fatigue Properties

**Figure 11:**
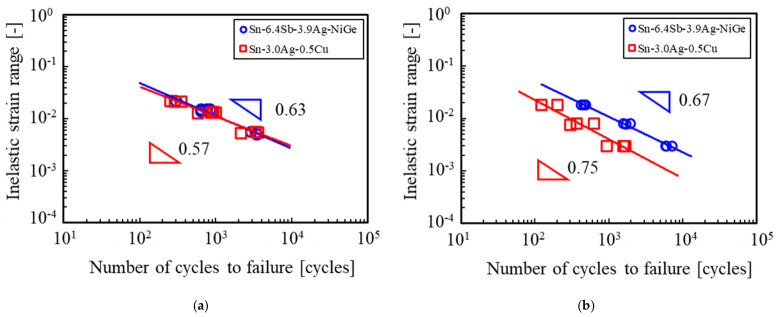
Relationship between the inelastic strain range and the fatigue life (**a**) at room temperature and (**b**) at 175 °C.

Figure 11 shows the relationship between the inelastic strain range obtained from the hysteresis loop in the fatigue test and the number of cycles to failure obtained from the fatigue test for the as-cast specimen. In the low-cycle fatigue test, in which inelastic deformation becomes dominant, the relationship between the inelastic strain range and the number of cycles to failure follows the Manson–Coffin equation, as shown in Equation (4) [25,26,27].

Δ*ε*_p_ = C_p_·(*N*_f_)^−α^ (4)

where Δ*ε*_p_ is the inelastic strain amplitude, C_p_ is the fatigue ductility coefficient, *N*_f_ is the fatigue life, and α is the fatigue ductility exponent. As shown in Figure 11a, the low-cycle fatigue properties of Sn-6.4Sb-3.9Ag-0.25Ni-0.003Ge and Sn-3.0Ag-0.5Cu at room temperature are comparable, and their fatigue ductility exponents are also similar at 0.63 and 0.57, respectively. By contrast, the results at 175 °C (Figure 11b) show that the regression line for Sn-3.0Ag-0.5Cu shifts downward, confirming the superior fatigue properties of Sn-6.4Sb-3.9Ag-0.25Ni-0.003Ge. Furthermore, Equations (5) and (6) show the Manson–Coffin relationships fitted to the experimental data at 175 °C for the Sn-6.4Sb-3.9Ag-0.25Ni-0.003Ge and Sn-3.0Ag-0.5Cu alloys, respectively.

Δ*ε*_p_ = 1.16·(*N*_f_)^−0.67^ (5)

Δ*ε*_p_ = 0.70·(*N*_f_)^−0.75^ (6)

The fatigue ductility exponent is larger for Sn-3.0Ag-0.5Cu (0.75) than for Sn-6.4Sb-3.9Ag-0.25Ni-0.003Ge (0.67). In the Manson–Coffin equation, the fatigue ductility exponent represents the sensitivity of a material’s fatigue life to applied inelastic strain. A steeper negative slope in Figure 11 demonstrates that even minimal inelastic strain applied during low-cycle fatigue decreases the fatigue life of Sn-3.0Ag-0.5Cu.

In low-cycle fatigue, the fatigue ductility exponent α is closely related to the cyclic strain-hardening exponent. It has been reported that the fatigue ductility exponent increases as the cyclic strain-hardening exponent decreases [13,28]. Additionally, microstructural changes, such as IMC coarsening, significantly influence the cyclic strain-hardening exponent [29]. In dispersion-strengthened solder alloys, the coarsening of IMC driven by temperature and inelastic strain reduces the cyclic strain-hardening exponent, which increases the fatigue ductility exponent. Therefore, in Sn-3.0Ag-0.5Cu, where significant IMC coarsening occurred under the high-temperature (175 °C) environment, the increase in the fatigue ductility exponent suggests a corresponding degradation of fatigue life. By contrast, in Sn-6.4Sb-3.9Ag-0.25Ni-0.003Ge, the suppressed IMC coarsening maintained the cyclic strain-hardening exponent. As a result, the subsequent rise in the fatigue ductility exponent was prevented, suggesting a strong correlation between the stabilization of IMC morphology and the trend of the fatigue ductility exponent. However, a noticeable shift in the intercept of the Manson–Coffin plots was observed between the two alloys, where Sn-6.4Sb-3.9Ag-0.25Ni-0.003Ge exhibited a higher fatigue ductility coefficient, C_p_. This difference in the intercept is associated with alterations in the dominant deformation mechanisms induced by the complex alloying elements. Although detailed microstructural verification of the intercept is outside the scope of this study, it warrants further systematic investigation in future work.

## 4. Conclusions

In this study, the coarsening behavior and microstructural changes in Ag_3_Sn in a Sn-6.4Sb-3.9Ag-0.25Ni-0.003Ge solder during high-temperature aging were investigated. The data were compared with those of a conventional Sn-3.0Ag-0.5Cu solder. The results are as follows:

(1) In the as-cast microstructure of Sn-6.4Sb-3.9Ag-0.25Ni-0.003Ge, SbSn phases are dispersed in the β-Sn matrix. Although Sb dissolves in the β-Sn phase and SbSn phases disappear as the temperature rises, some of the SbSn phases aggregate.
(2) The activation energy for the coarsening of Ag_3_Sn in Sn-6.4Sb-3.9Ag-0.25Ni-0.003Ge was 45 kJ/mol, which is comparable to that of Sn-3.0Ag-0.5Cu, demonstrating that the temperature dependence of the Ostwald ripening rate is equivalent.
(3) In Sn-6.4Sb-3.9Ag-0.25Ni-0.003Ge, the solubility limit of Ag in the β-Sn phase decreases due to the solid solution of Sb in the β-Sn phase at high temperatures, which delays the coarsening of Ag_3_Sn.
(4) The relationship between the inelastic strain range and the cycles to failure obtained from the low-cycle fatigue tests for each alloy follows the Manson–Coffin equation. Moreover, it was suggested that the suppression of Ag_3_Sn coarsening inhibited the decrease in the fatigue ductility exponent, thereby improving the fatigue resistance at 175 °C.

## Figures and Tables

**Table 1:** Melting properties of alloys used in this study.

Chemical Compositions [mass%]	Solidus Temperature [°C]	Liquidus Temperature [°C]
Sn-6.4Sb-3.9Ag-0.25Ni-0.003Ge	229	234
Sn-3.0Ag-0.5Cu	218	219

## Data Availability

The original contributions presented in this study are included in the article. Further inquiries can be directed to the corresponding author.
